# Multimodal profiling of immune responses reveals innate-adaptive immune imbalance in human bornavirus encephalitis

**DOI:** 10.1186/s40478-026-02319-6

**Published:** 2026-05-14

**Authors:** Nicola Jungbäck, Przemyslaw Grochowski, Daniel Hieber, Moritz Dinser, Zuzanna Mielewczyk, Thomas Pfefferkorn, Birgit Muntau, Thomas Richter, Georg Rieder, Antonios Bayas, Klaus Hirschbühl, Bruno Märkl, Patrick Adam, Dennis Tappe, Friederike Liesche-Starnecker

**Affiliations:** 1 Faculty of Medicine, Institute of Neuropathology, University Medical Centre Ulm, Ulm University, Albert-Einstein-Allee 23, 89081 Ulm, Germany; 2 Pathology, Medical Faculty, University of Augsburg, Stenglinstraße 2, 86156 Augsburg, Germany; 3 Institute DigiHealth, Neu-Ulm University of Applied Sciences, Wileystraße 1, 89231 Neu-Ulm, Germany; 4 Institute of Medical Data Science, University Hospital Würzburg, Josef-Schneider-Straße 2, 97080 Würzburg, Germany; 5 Department of Neurology, Ingolstadt Hospital, Krumenauerstraße 25, 85049 Ingolstadt, Germany; 6 Bernhard Nocht Institute for Tropical Medicine, National Reference Centre for Tropical Pathogens, Bernhard-Nocht-Straße 74, 20539 Hamburg, Germany; 7 Pathology, Lilienweg 12, 83022 Rosenheim, Germany; 8 Department of Neurology, InnKlinikum, Vinzenz-Von-Paul-Straße 10, 84503 Altötting, Germany; 9 Department of Neurology and Clinical Neurophysiology, Faculty of Medicine, University of Augsburg, Stenglinstraße 2, 86156 Augsburg, Germany; 10 Hematology and Oncology, Medical Faculty, University Hospital of Augsburg, Stenglinstraße 2, 86156 Augsburg, Germany; 11 Pathology, Levelingstraße 21, 85049 Ingolstadt, Germany; 12 Institute of Pathology, School of Medicine and Health, Technical University of Munich, Trogerstraße 18, 81675 München, Germany

**Keywords:** BoDV-1, Encephalitis, Neurotropic virus, Immunopathogenesis, Transcriptome

## Abstract

Human bornavirus encephalitis (BVE) is a rare, emerging and fatal zoonotic disease mainly caused by the Borna disease virus 1 (BoDV-1), a non-cytolytic RNA virus. Despite increasing recognition, the immunopathogenesis of human BoDV-1 infection remains insufficiently characterised. Complete coronal and sagittal brain sections from four fatal BoDV-1 cases were analysed using digitised immunohistochemistry to quantify viral distribution and tissue responses. Transcriptome-based analyses characterised local immune cell profiles in relation to viral loads measured by RT-qPCR. BoDV-1 viral loads varied substantially between cases but showed region-specific enrichment in the basal ganglia and hippocampus, correlating with lymphocyte presence and reactive microglia and astrocytes. Immune cell deconvolution revealed viral load-dependent modulation dominated by innate immune and glial populations, including metabolic and reactive astrocyte states, IFNγ-responsive microglia, and dendritic cells, macrophages, neutrophils, basophils, and CD8⁺ T cells. This was accompanied by induction of interferon-stimulated genes, antigen presentation, protein synthesis, and oxidative stress pathways, with a transcriptional signature resembling non-lytic viral and autoimmune-like neuroinflammatory conditions rather than lytic infections. These findings support a model of BoDV-1 encephalitis characterised by a prominent innate immune response and comparatively limited adaptive immune signatures. This imbalance might potentially contribute to impaired viral clearance and extensive tissue damage, a possible relationship that warrants further investigation.

**Supplementary Information:**

The online version contains supplementary material available at 10.1186/s40478-026-02319-6.

## Introduction

Human bornavirus encephalitis (BVE) is a rare but mostly fatal disease. While the main causative pathogen, the Borna disease virus 1 (BoDV-1), has been known to infect animals for decades, the first confirmed human cases were only identified in 2018 [18]. Since then, BoDV-1 has been recognised as a significant zoonotic pathogen in the endemic areas in Southern and Eastern Germany [4], reflecting its association with shrew reservoirs that are confined to geographically limited habitats [3]. Approximately five to ten new human cases are confirmed each year, with a cumulative total of around 50 known cases to date [14]. Clinically, BoDV-1 infection often begins with nonspecific, flu-like symptoms, followed by rapid neurological deterioration with altered consciousness, seizures, and motor deficits. In most cases, the disease progresses swiftly, leading to death within a few weeks after symptom onset [7, 14, 24]. Survivors typically suffer from severe long-term sequelae, including profound neurological impairment such as a vegetative state (apallic syndrome). The combination of high case fatality, rapid progression, and diagnostic challenges underscore the urgent need for increased clinical awareness and further therapeutic research, while also positioning BVE as valuable model for studying the pathogenesis of highly neurotropic viruses in general.

Our initial neuropathological investigations described the first histological characteristics of BVE in six human autopsy cases. The viral distribution pattern showed prominent involvement of the basal ganglia with histological definition as a sclerosing panencephalitis with lymphocytic infiltration, microglial nodules, and intranuclear eosinophilic inclusions [8]. Subsequent findings revealed the presence of BoDV-1 in endothelial cells, observed under certain conditions and linked to vascular injury caused by hypoxic stress [9]. Finck et al*.* extended the understanding of BVE by correlating magnetic resonance imaging (MRI) findings with histopathological changes in a separate set of cases. They identified a reproducible pattern of radiological changes, beginning in the caudate nucleus and insular cortex, followed by involvement of the limbic system and brainstem [5].

Although the exact immunopathological mechanisms remain unclear to date, evidence suggests that BoDV-1 is non-cytolytic, with tissue injury primarily immune-mediated, involving CD4⁺ and CD8⁺ T cells, as well as reactive astrocytes, which are increasingly recognised as active contributors to disease progression [12, 15]. In selected cases, immunosuppressive treatment has been associated with prolonged survival, underscoring the potential role of immune modulation in disease management [7, 15]. Serum and cerebrospinal fluid analysis from ten individuals with BVE demonstrated a predominantly proinflammatory cytokine profile, indicating sustained recruitment of immune cells. Such an inflammatory milieu may also disrupt astrocyte function, potentially resulting in neuronal excitotoxicity [15].

Although individual immunopathological features of BVE have been described, the spatial viral distribution within the human brain and the neuroimmune responses remain insufficiently characterised. To address these gaps, we systematically analysed BoDV-1 dissemination in fully embedded, anatomically preserved brain cross-sections, and performed in-depth, transcriptome-based profiling of host neuroimmune responses. We hypothesised that BoDV-1 infection induces a distinct neuroinflammatory response shaped by viral tropism and local tissue context. Beyond advancing the understanding of BVE pathogenesis, our findings present a methodological approach, which can be applied to investigate host–pathogen interactions in other neurotropic viral encephalitides.

## Material and methods

### Material

Brain autopsy material from four individuals (ages 39—71 years; median 68.5 years; all female) who died of BVE between 2022 and 2024 was included. Cases 3 and 4 were previously described [2, 24], whereas Cases 1 and 2 have not been published yet.

For Cases 1 and 2, complete sagittal sections of the right hemisphere and complete coronal sections of the left hemisphere were prepared. Case 3 included a right sagittal section; Case 4 a coronal section through both hemispheres to assess viral distribution symmetry. Cross-sections were subdivided into approximately equally sized blocks and were formalin fixed and paraffin embedded (FFPE) in standard histology cassettes, enabling subsequent histological processing and reconstruction of whole-brain slices. In total, 157 FFPE blocks were included in the analyses (median 37 blocks per case; range 31–52). Control tissue comprised of two FFPE blocks (left thalamus and frontal cortex) from a 64-year-old woman who died of toxic cardiocirculatory failure in the setting of advanced metastatic breast carcinoma and pleural effusion.

Ethical approval was obtained from the ethics committee of the Ludwig-Maximilians-University ethics committee, responsible for Augsburg University Hospital (approval no. 23-0267).

### General study design and workflow

**Fig. 1:**
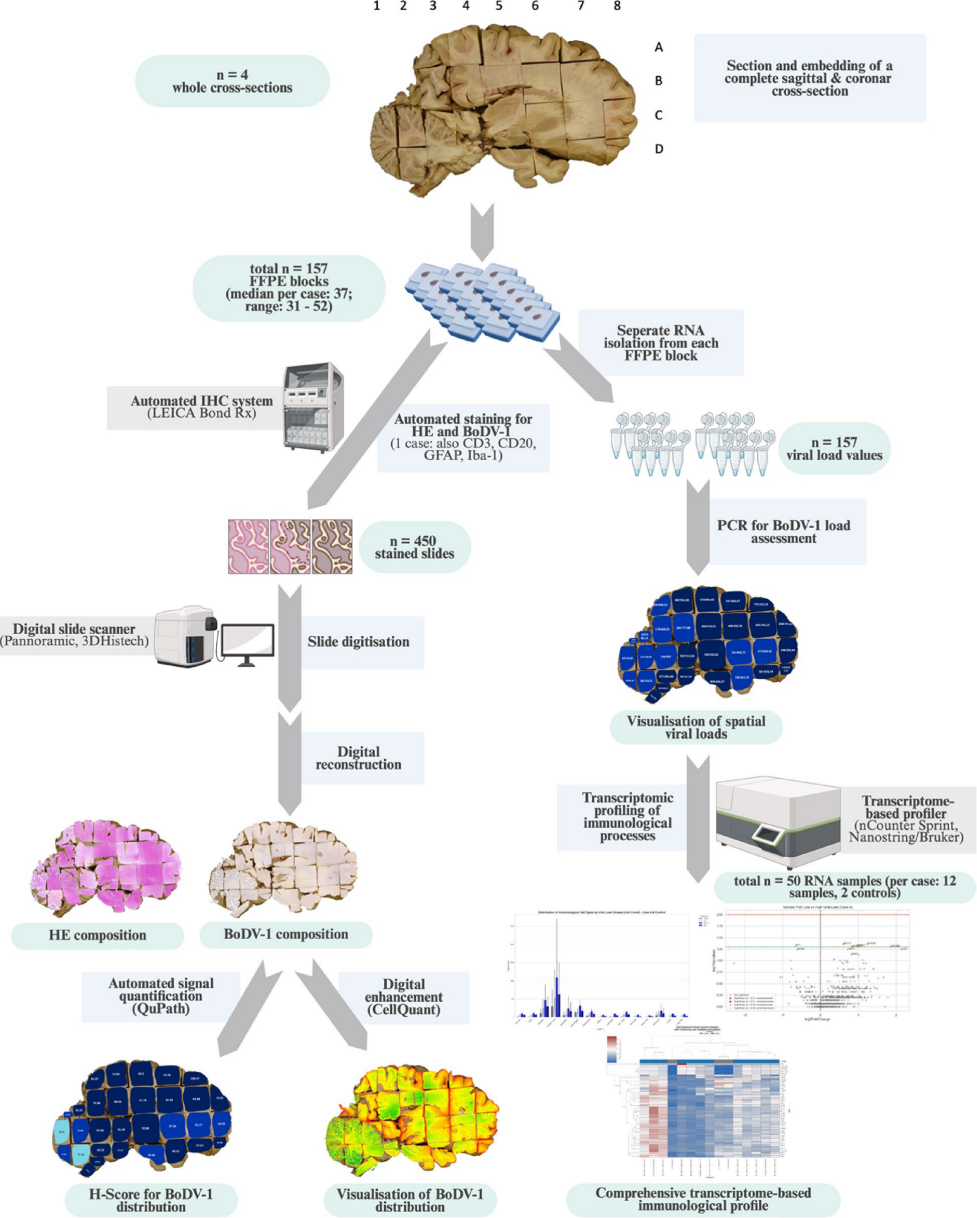
Outline of the general project framework

As outlined in Fig. 1, all FFPE blocks were stained with hematoxylin and eosin (HE) and BoDV-1 immunohistochemistry (IHC) using automated staining systems (HE: Tissue-Tek Prisma, Sakura, Japan; IHC: Leica Bond RX, Leica Biosystems, Germany). In Case 4, additional immunostainings for T cell marker CD3, B cell marker CD20, microglia marker Iba-1, and astrocytic marker GFAP were performed. A total of 450 slides were prepared and digitised (scanner Pannoramic II, 3DHISTECH, Hungary).

Sagittal and coronal cross-sections were digitally reconstructed. Based on BoDV-1 immunoreactivity, automated signal quantification analysis using the QuPath software [1] was performed, generating a histopathological (H-)Score for each FFPE block. Additionally, CellQuant software (3DHISTECH, Hungary) was used for enhanced visualisation and spatial assessment of BoDV-1 signal distribution. RNA was separately extracted from all blocks to determine BoDV-1 viral loads. For transcriptomic profiling, 12 FFPE blocks per case (covering low, medium, and high viral loads; total: 50 samples, incl. two controls) were analysed using the nCounter Sprint Profiler (NanoString/Bruker, United States) to characterise immunological gene expression profiles. The resulting data were subjected to statistical analysis using custom Python (Python Software Foundation, United States) workflows. Details on all methods are provided in the Supplementary Material.

## Results

### Regional BoDV-1 viral load profiling reveals marked interindividual variability but consistent hotspots in deep brain structures

**Fig. 2:**
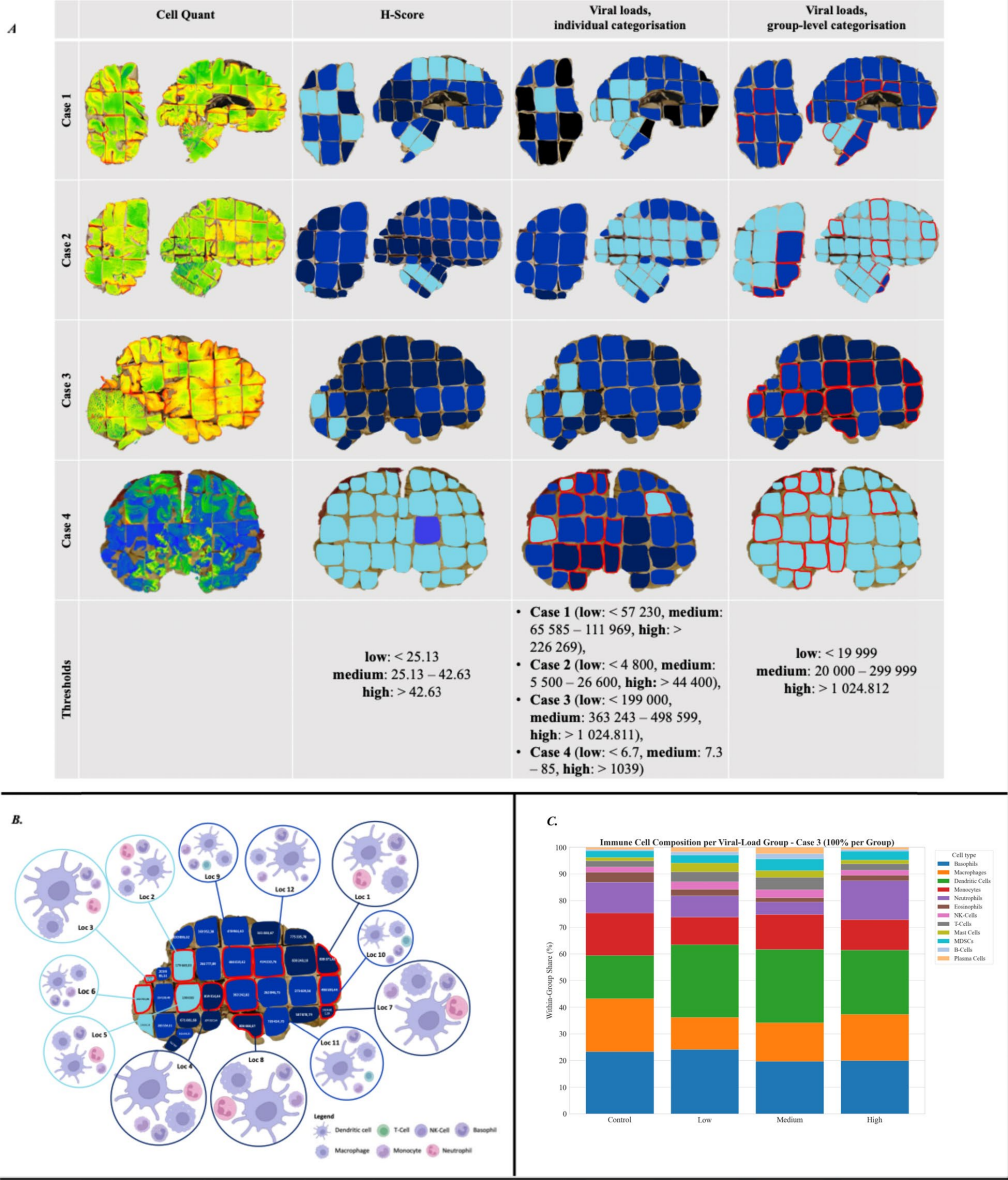
Integrated spatial analysis of viral distribution and immune cell landscape in BoDV-1-infected human brain tissue. **A** Whole brain sections were evaluated using CellQuant-based BoDV-1 distribution visualisation, semi-quantitative H-Score assessment of BoDV-1 immunohistochemistry, and viral load quantification. Colour coding for H-Score and viral loads reflects the intensity of BoDV-1 detection: dark blue indicates high levels, royal blue medium levels, and light blue low levels. Due to marked interindividual variability, viral load classification was performed within each individual case (3rd column) and across all cases (4th column). Blocks marked in red indicate those selected for downstream transcriptomic profiling. Threshold values for viral loads in copies/ng RNA. **B** Sagittal brain section of Case 3 illustrating regional viral load distribution. Areas with high viral load are shown in dark blue, medium viral load in royal blue, and low viral load in light blue. The size of each depicted immune cell type reflects its relative abundance in the respective region, as determined by transcriptomic analysis. Only the five most abundant immune cell population per location are illustrated. **C** Overall immune cell composition stratified by viral load group for Case 3 is summarised

Coronal and sagittal brain sections were analysed using three complementary approaches (Fig. 2A): BoDV-1 immunoreactivity was assessed via CellQuant-based digital visualisation and semi-quantitative H-Scoring using QuPath (see Supplementary Material Table 1). In parallel, BoDV-1 RNA levels were determined by quantitative reverse transcription polymerase chain reaction (RT-qPCR) across all FFPE blocks. For the standardised anatomical assignment of tissue blocks see Supplementary Material Fig. 1.

Viral loads were subsequently classified into low, medium, and high categories, first within each individual case and then across all cases. Due to marked interindividual variability, distinct thresholds were applied for H-Score assessment, individual viral load classification, and group-level categorisation.

Mean viral loads differed interindividually by several orders of magnitude with ranges from 170.37 copies(c)/ng RNA in Case 4 to 462 711.72 c/ng RNA in Case 3. Case 1 (94 998.83 c/ng RNA) and Case 2 (9 343.39 c/ng RNA) showed intermediate values. Across all cases, FFPE blocks containing the basal ganglia, hippocampus, thalamus, and brainstem consistently showed higher viral loads. This regional pattern was mirrored by immunohistochemistry: H-Score analysis revealed the highest mean score in Case 3 (54.35), and the lowest in Case 4 (6.11), with intermediate values in Cases 1 (36.23) and 2 (46.77; Supplementary Material Tables 2 and 3).

BoDV-1 RNA levels and antigen detection showed weak but statistically significant positive correlations (Pearson’s ρ = 0.317, *p* < 0.001; Spearman’s ρ = 0.244, *p* = 0.002), consistent with a parallel distribution of viral RNA and immunoreactivity for BoDV-1 nucleoprotein. Given the expected non-normal distribution and potential influence of outliers in tissue-based measurements, both Pearson and Spearman correlations were calculated to assess linear and rank-based associations.

### Bilateral viral load comparison shows symmetric distribution in case 4

In Case 4, paired measurements of viral load revealed a highly symmetric distribution between both hemispheres. Pearson correlation analysis demonstrated a strong positive linear correlation (ρ = 0.951, *p* < 0.001) between the respective FFPE blocks, supporting a symmetric viral spread. This was corroborated by a paired t-test, which showed no significant difference between hemispheres (t = − 0.974, *p* = 0.3480).

### Correlation analysis identifies coordinated t cell, microglial, and astrocytic responses to BoDV-1 RNA levels

For Case 4, an intra-case correlation analysis was performed to explore associations between BoDV-1 levels and cellular immune markers. Spearman correlation coefficients were calculated for BoDV-1 RNA levels, and the expression levels of CD3 (T cells), CD20 (B cells), Iba-1 (microglia), and GFAP (astrocytes). The analysis revealed several significant correlations (Supplementary Material Fig. 2). BoDV-1 RNA levels showed a moderate positive correlation with CD3-positive cells (ρ = 0.587, *p* < 0.001) and correlated moderately with Iba-1 (ρ = 0.502, *p* = 0.003), and GFAP (ρ = 0.406, *p* = 0.019), according to the classification by Schober et al*.* [20]*.* Additionally, all pairwise combinations of CD3, Iba-1, and GFAP showed significant positive correlations (CD3/GFAP: ρ = 0.493, *p* = 0.003; CD3/Iba-1: ρ = 0.403, *p* = 0.018; Iba-1/GFAP: ρ = 0.480, *p* = 0.004). These correlations were conducted for Case 4, in which a more extensive immunohistochemical analysis was performed (see Supplementary Material Fig. 3).

**Fig. 3:**
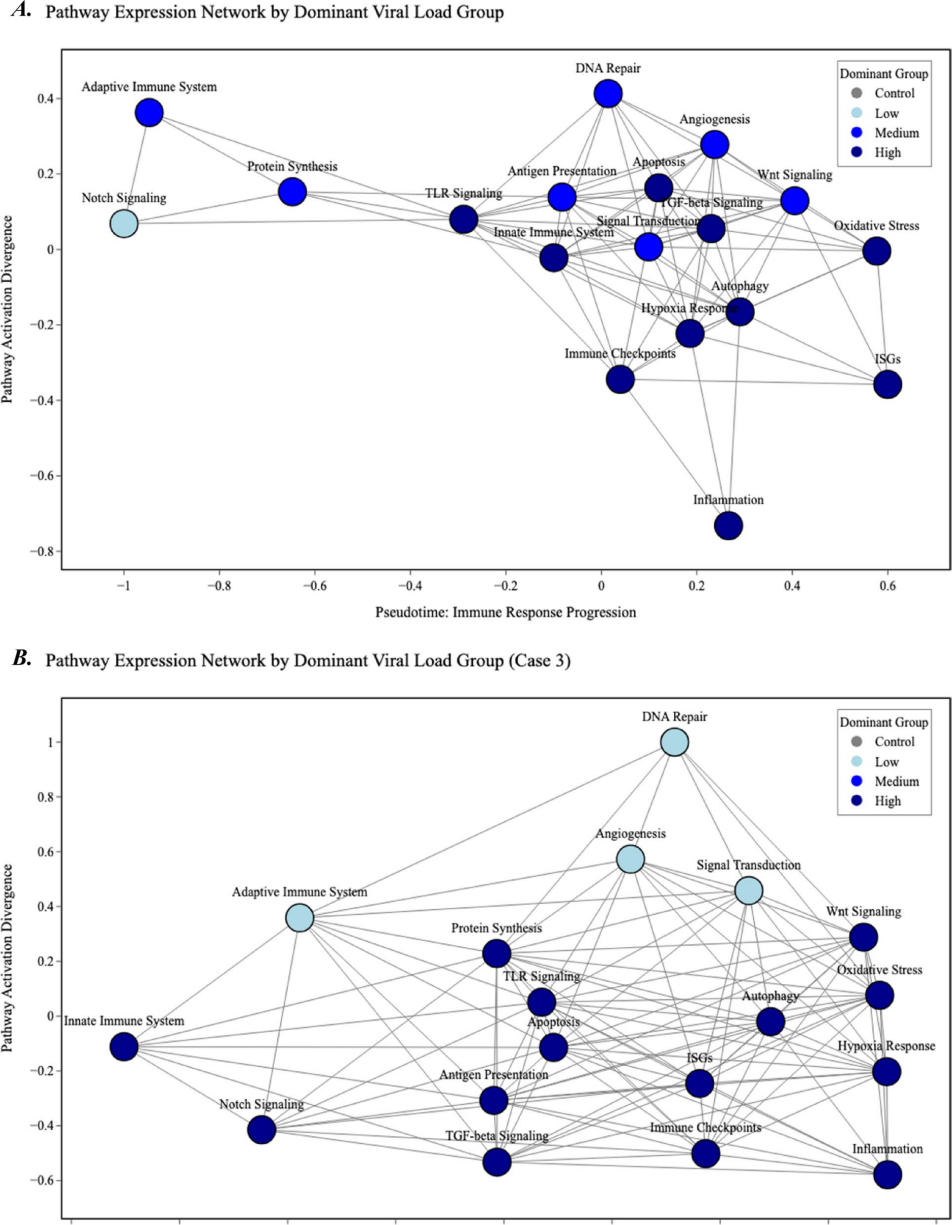
Expression-based pathway network across all viral load conditions. Each node represents a biological pathway, coloured by the viral load group in which it shows highest relative. Edges connect pathways with highly similar expression patterns across conditions, defined by Pearson correlation (r > 0.8). Node positions reflect functional proximity derived from graph layout, forming a pseudotemporal map of immune response dynamic, including all cases (**A**), and only Case 3 (**B**)

### Transcriptomic immune cell mapping suggests viral load-dependent modulation of immune and glial populations

To systematically assess the relationship between viral burden and immune activation, all tissue samples were stratified into three groups (BoDV-1_high_, BoDV-1_medium_, BoDV-1_low_) based on viral RNA copy numbers (thresholds listed in Fig. 2A). A non-infected control served as reference. In total, 50 tissue samples were selected for transcriptome-based profiling using the nCounter platform, providing high-resolution insights into the immune microenvironment across varying viral loads in different brain areas. Immune cell composition was inferred by transcriptomic deconvolution using cell-type-specific gene signatures and pathways, covering not only brain-specific cell types such as astrocytes and microglia, but also immune cells attracted to the site of inflammation. The aim was to determine whether distinct immune cell subsets are differentially enriched across varying levels of viral loads.

The marked interindividual differences in viral burden necessitated an additional intra-case evaluation for Case 3, which exhibited by far the highest viral RNA levels. As shown in Fig. 2A, the thresholds for viral load categories were accordingly adapted.

Across all BoDV-1-infected brain samples, a clear viral load-dependent modulation of immune cell populations was observed (Supplementary Material Fig. 4A). Dendritic cells emerged as the most abundant population under all viral load groups. Macrophages were the second most prominent population, which seems consistent with their role in phagocytic clearance and cytokine release. Basophils and neutrophils showed moderate enrichment, which likely reflects a secondary influx in response to tissue damage and inflammation. Other immune subsets, including monocytes, natural killer (NK) cells, and T cells, exhibited a similar abundance in response. Plasma cells, eosinophils, B cells, mast cells, and myeloid-derived suppressor cells, were only sparsely detected, indicating a limited role in the dominant inflammatory response.

**Fig. 4:**
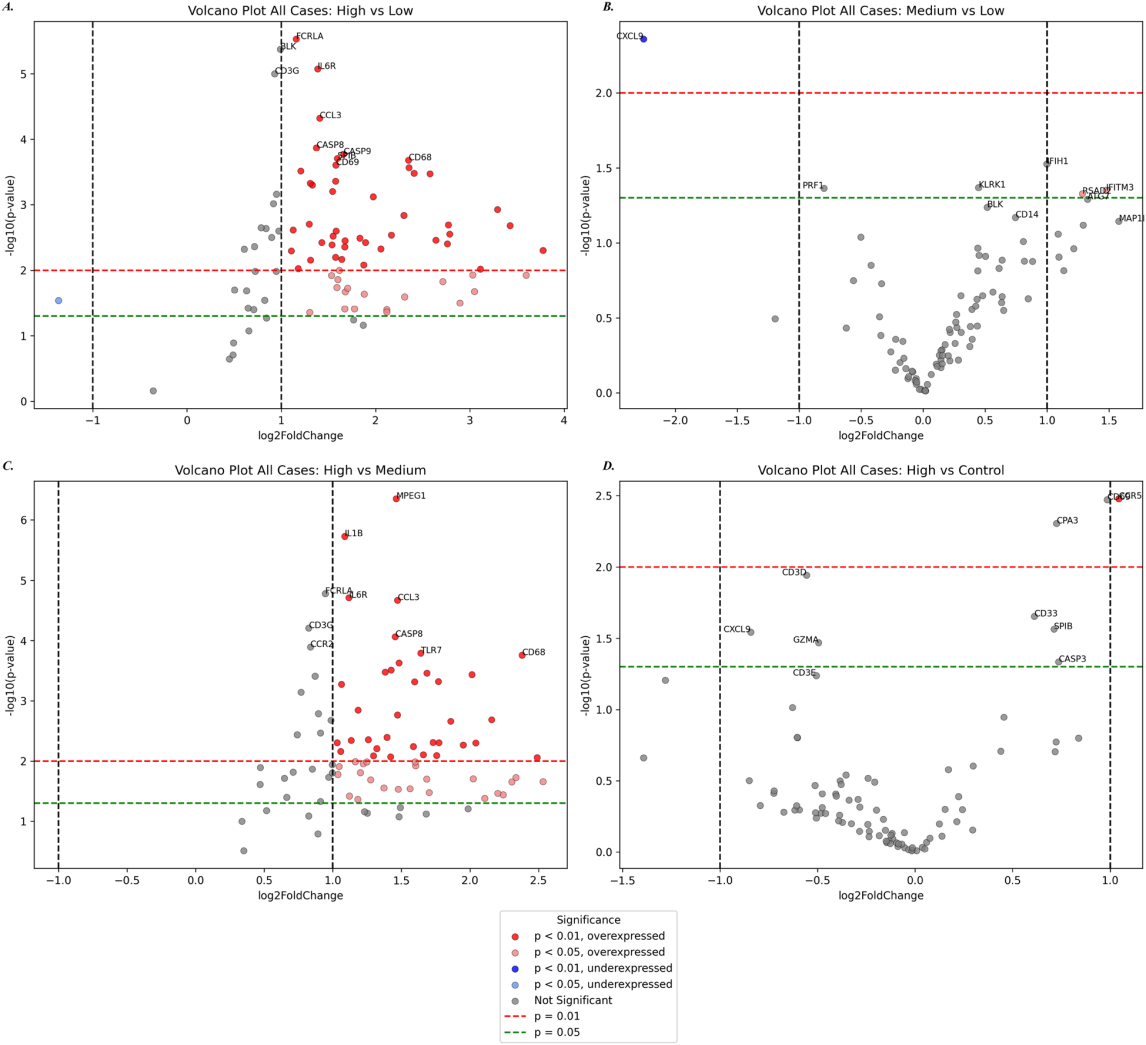
Differential gene expression in BoDV-1-infected brain regions across viral load stages and in comparison, to non-infected control tissue. Volcano plots display pairwise comparisons between brain regions with differing BoDV-1 viral loads and control tissue. Genes significantly upregulated (red) or downregulated (blue) are highlighted according to significance thresholds (*p* < 0.05 and *p* < 0.01)

Although T cells were not the most dominant immune population, they play a crucial role in the immune response to BoDV-1 infection, and, exhibiting cuffing around blood vessels and tissue infiltration are a histopathological hallmark of encephalitis. A more detailed analysis of T cell subsets revealed that CD8⁺ T cells were the most abundant across all BoDV-1 viral load groups, followed by CD4⁺ and memory T cells (Supplementary Material Fig. 4B). The distribution followed a similar pattern, with the low groups exhibiting slightly higher values than the medium viral load groups, particularly for the CD8⁺ T cells. For CD4⁺ and memory T cells, the distribution was relatively even across all groups.

A closer look at the astrocyte and microglia subsets revealed a similar distribution pattern with predomination of the disease-associated subtype (Supplementary Material Fig. 4* C/D*). In astrocytes, this was followed by the metabolic and reactive subtypes, while in microglia, the interferon-γ-induced subtype ranked second. These findings suggest a strong association between the viral load and the activation of specific immune and glial cell subsets.

To minimise variability between individuals and confirm the findings under more controlled conditions, a within-case analysis was repeated for Case 3. Notably, the results closely resembled the patterns seen across all BoDV-1 viral load groups, supporting the homogeneity of the observed immune profile. Dendritic cells again emerged as the predominant population, irrespective of local viral load (Fig. 2B/C), followed by macrophages. Neutrophils and basophils were also repeatedly detected, indicating innate immune recruitment in response to infection or tissue damage. Although monocytes, NK cells, and T cells appeared less frequently, they were consistently present (Supplementary Material Fig. 5A).

**Fig. 5:**
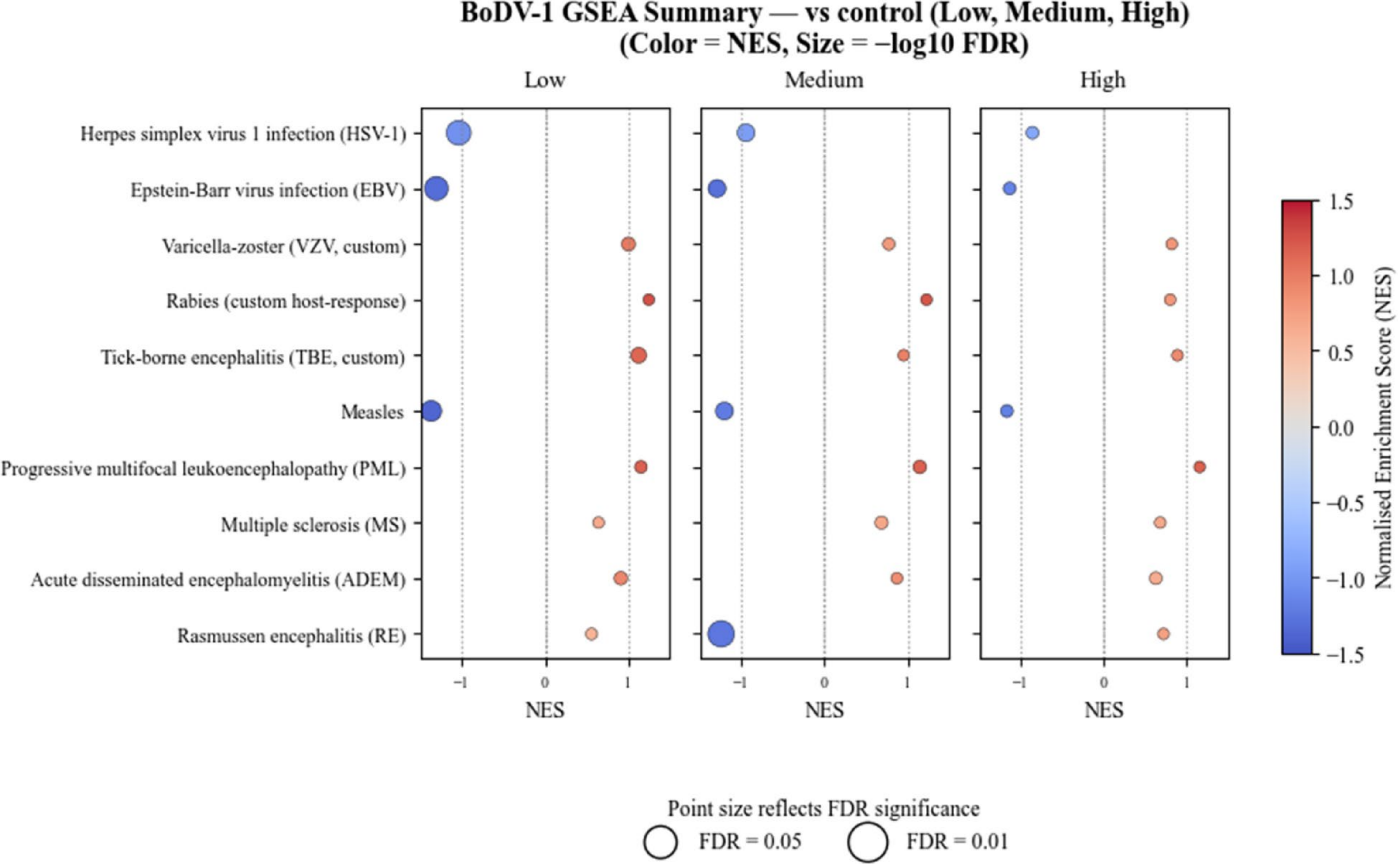
KEGG Enrichment Analysis with GSEA Summary. GSEA using KEGG pathways was performed across BoDV-1 viral load groups. Dot size represents significance of pathway enrichment, and the NES the strength and direction of enrichment. KEGG-defined host antiviral response pathways capture conserved immune programs that are commonly activated during viral infections and autoimmune inflammatory diseases, reflecting shared antiviral mechanisms. A custom gene set comprising antiviral and neuronal stress-associated genes described in herpesvirus (HSV-1, EBV, VZV), rabies, TBE, and measles infections, as well as inflammatory and demyelinating conditions including PML, MS, ADEM, and RE, was included to enable comparative analyses across neurotropic viral and immune-mediated CNS pathologies

A more detailed analysis of T cells, astrocytes, and microglia confirmed this trend: CD8⁺ T cells were again by far the most dominant subset and their relative abundance slightly decreased in BoDV-1_medium_ regions (Supplementary Material Fig. 5B), accompanied by a marked accumulation of disease-associated astrocytes (Supplementary Material Fig. 5C) and microglia (Supplementary Material Fig. 5D).

To validate the results of transcriptionally inferred cellular composition, we performed correlation analyses with quantitative immunohistochemical data. A strong and statistically significant correlation was observed for CD3, supporting the robustness of the inferred T-cell signal. Microglia-associated markers showed a moderate association. In contrast, CD20 and astrocyte-associated signals did not reach statistical significance (Supplementary Material Fig. 6).

### Pathway and pseudotime analysis reveal load-dependent innate activation and limited adaptive immune engagement

Across all BoDV-1-infected brain samples, transcriptomic patterns across different viral load levels were dominated by innate immune signalling pathways, with progressive upregulation of interferon-stimulated genes (ISGs), antigen presentation, protein synthesis, and stress-response pathways (Supplementary Material Fig. 7A) indicating dose-dependent activation of type I interferon signalling via pattern recognition receptors, a defining feature of acute antiviral immunity [19].

The within-case analysis of Case 3 (Supplementary Material Fig. 7B) mirrored these patterns, showing the same ISG- and antigen-presentation-driven response with similar distribution across viral load groups, despite minor shifts in the relative ranking of pathways.

The pathway-level expression network revealed a structured immune response aligned with viral load (Fig. 3A). In BoDV-1 encephalitis, pathway clustering across pseudotime (an inferred temporal progression of biological states) reflected a progression from early cellular maintenance to advanced immune activation. Low viral load tissue was dominated by Notch signalling, which occupied an isolated position at the early end of the pseudotime axis. Medium viral load samples were associated with DNA repair, protein synthesis, signal transduction, angiogenesis, and Wnt signalling, forming a central cluster indicative of early-to-intermediate cellular activation. In contrast, high viral load samples displayed dense clustering of stress- and immune-related pathways.

Of note, adaptive immune pathways were located at the interface between medium and high viral load clusters, without forming a clearly distinct pattern. Control samples did not form a dominant cluster, underscoring their limited role in the overall immune activation profile.

In Case 3, low and high viral load samples dominated the expression landscape (Fig. 3B). Low viral load was associated with early response pathways such as DNA repair, adaptive immunity, and angiogenesis, whereas high viral load corresponded to robust activation of innate immunity, oxidative stress, inflammation, and immune checkpoints. Notably, adaptive immune signalling did not form a coherent cluster but instead showed a more diffuse spatial distribution despite persistent viral presence.

### Gene expression analysis shows threshold-dependent activation of innate and inflammatory pathways

Transcriptomic profiling across all four cases revealed viral load-dependent immune signatures. Differential gene expression was most pronounced in comparisons involving BoDV-1_high_ samples (vs. BoDV-1_medium_ and vs. BoDV-1_low_; Fig. 4) and suggests that immune activation appears more pronounced at higher viral loads. In BoDV-1_high_ tissue, the marked upregulation of CD68, IL1B, and CCL3 indicated pronounced microglial activation and proinflammatory signalling. The consistent increase in CASP8 expression further suggested an engagement of inflammatory cell death pathways under high viral load conditions. Compared to uninfected controls, BoDV-1_high_ samples further showed increased expression of CCR5, supporting an enhanced chemokine-mediated immune cell recruitment to the CNS [22]. Interestingly, CXCL9, a pro-inflammatory chemokine involved in T cell recruitment, was significantly underexpressed in BoDV-1_medium_ compared to BoDV-1_low_ samples (Fig. 4B).

In Case 3, BoDV-1_high_ samples exhibited pronounced transcriptional changes consistent with strong antiviral immune activation (Supplementary Material Fig. 8). Most significantly upregulated genes included CD68 (activated microglia/macrophages), TLR2 (sensor of viral ligands), OAS1 (interferon-stimulated antiviral effector), and CXCL10 (T cell recruiting chemokine), reflecting coordinated activation of innate immune sensors and CNS-resident effector cells. Upregulation of FCGR3 supports a role for antibody-dependent innate mechanisms [11].

Comparison between medium and low viral load revealed only minor changes. Compared to controls, BoDV-1_high_ samples showed increased CASP3 and CCR5, indicating enhanced cytotoxicity and leukocyte recruitment [22].

### pathway-based gene set analysis reveals upregulation of non-lytic viral and autoimmune-like signatures

A Kyoto Encyclopedia of Genes and Genomes (KEGG)-based custom gene set enrichment analysis (GSEA), including neurotropic, inflammatory, and non-pathogen-associated encephalitic reference sets, revealed a structured and category-specific transcriptional response across BoDV-1 viral load groups (Fig. 5). Compared to control tissue, the signatures for herpes simplex virus 1 (HSV-1), measles and Epstein-Barr virus were downregulated across all groups. In contrast, the signatures for the neurotropic tick-borne virus, rabies virus (RV), varicella-zoster virus and JC-virus (causative agent of progressive multifocal leukoencephalopathy) were uniformly upregulated. Interestingly, signatures for autoimmune diseases, such as multiple sclerosis and acute disseminated encephalomyelitis also showed upregulation.

## Discussion

BVE is an emerging, severe and mostly fatal inflammatory disease of the central nervous system without any established treatment regimen. Neuropathological studies describe a lymphocyte-rich panencephalitis with widespread lesions showing intranuclear inclusions in neurons and glial cells, microglial nodule formation, astrocyte activation, and severe tissue damage primarily driven by a strong proinflammatory immune response. Although involvement of CD4^+^/CD8^+^ T cells, microglial and astrocytic activation, and proinflammatory cytokine production has been described [8, 12, 15], the associated neuroimmune responses in the human brain remain poorly understood. This study aimed to address this gap through anatomically resolved viral mapping and transcriptome-based immune profiling.

Our results show that BoDV-1 viral loads varied markedly between individuals. High viral burdens in the basal ganglia, hippocampus, thalamus, and brainstem are consistent with previous reports [5, 6, 8]. Notably, in contrast to earlier studies based on highly variable sampling strategies [5, 8, 9], the use of a more standardised sampling approach in our cohort allows for improved comparability of anatomical regions. However, differences in sectioning planes and anatomical levels, which were intentionally incorporated to capture broader anatomical representation, should be taken into account when interpreting regional patterns. Additionally, potential confounding effects related to systemic disease and end-stage conditions may influence the interpretation of control tissues. Correlation between BoDV-1 RNA levels and immune cell and glial markers suggest an association of viral replication with T cell infiltration and reactive activation of microglia and astrocytes, features prominently observed in neuropathological examinations of BVE cases [8, 15]. Immune cell populations in BoDV-1-infected brain samples displayed distinct activation patterns linked to viral load levels. Local dendritic cells together with attracted macrophages, neutrophils, and basophils were the most abundant immune subsets based on computational inference, while CD8⁺ T lymphocytes dominated the adaptive compartment, consistent with Rauch et al*.* [15]. Although a moderate positive correlation between viral RNA levels and CD3⁺ T cell abundance was observed, stratification by viral load indicates a more nuanced, potentially stage-specific regulation of cytotoxic T cell responses. Concordance between BoDV-1 RNA and antigen levels was statistically significant, though weak, which may be attributable not only to variability in RNA integrity in FFPE tissue and differential stability of RNA and antigen, but also to biological factors such as differences in transcriptional activity and protein persistence.

The activation of ISGs, antigen presentation, protein synthesis, and oxidative stress pathways reflects a robust antiviral and cellular stress response. Notably, our data suggest a relative underrepresentation of adaptive immune signalling, which may reflect either a temporal delay in adaptive responses or active suppression mechanisms. This aligns with established processes of immune regulation, including T cell exhaustion, checkpoint inhibition, and pathogen-driven immune evasion [25]. Altogether, the findings indicate a tiered immune response in BVE, shifting from homeostatic and repair-associated states towards antiviral and stress-driven activation as viral loads increase. This constellation, characterised by a strong innate response alongside comparatively lower or suppressed adaptive immune signatures, may be associated with the presence of viral material despite pronounced inflammation and severe tissue destruction, and be relevant in the context of the observed fatal disease course. Importantly, these data do not contradict the established concept of immune-mediated pathology in BVE but highlight the complexity of immune regulation in advanced stages.

Immune activation appeared to increase with higher viral loads in our samples, that correlated with microglial activation, proinflammatory signalling, and cell death. Upregulation of CD68, IL1B, CCL3, CASP8, and CCR5, alongside downregulation of CXCL9 suggests a BoDV-1-associated immune environment characterised by altered T cell-associated signals and increased microglial activation, consistent with the presence of viral material in terminal-stage tissue. This finding highlights the need for a more detailed characterisation of microglia and their role during BoDV-1 infection.

When comparing with other neurotropic virus infections, such as HSV-1 and RV infection, overlapping immune features were observed in BVE. Both, BoDV-1 and HSV-1 infection show CD68 and CXCL10 upregulation [21, 23]. IL1B is also upregulated in RV-infected microglia [10]. However, notable differences emerged from our analyses. HSV-1 infection primarily leads to CCL2 and CCL5 upregulation [21], whereas BoDV-1 infection selectively induced CCL3. No human data on CXCL9 expression exist for HSV-1 or RV, with insights into its functional role during HSV-1 infection derived solely from animal models [26]. Additionally, BVE showed transcriptional activation of apoptosis-related genes (CASP8, CASP9, LC3A), while during HSV-1 infection, these are mainly regulated at the post-translational level; for RV, such data are lacking. These differences highlight virus-specific immunopathological mechanisms despite shared innate immune activation, suggesting that each virus engages distinct strategies to shape host responses, and influence disease outcome. This is supported by KEGG enrichment analyses, which showed limited overlap between BoDV-1 infection and transcriptional programs associated with cell-lytic viruses like HSV-1. This may explain the differences despite shared activation of innate immune pathways including oxidative stress responses, autophagy regulation, and antigen presentation [13, 16, 17]. In contrast, gene signatures associated with non-lytic neurotropic viruses, such as RV and tick-borne encephalitis virus, were also identified in BoDV-1 infection. Interestingly, gene sets related to autoimmune neuroinflammatory conditions were likewise enriched. This points to possible overlapping neuroimmune patterns and is consistent with previous clinical and neuropathological observations, including corticosteroid-responsive inflammation and lymphocytic involvement [6, 15, 24].

Despite the novelty of our investigations of the immunopathogenesis of BVE, some challenges and limitations have to be acknowledged. Human BVE is an extremely rare disease, thereby limiting the number of cases available for analysis. Moreover, heterogenous disease courses and treatment attempts, together with the terminal-stage autopsy material, differences in post-mortem interval, and tissue fixation time, limit generalisability.

However, even with these constraints, our data offer valuable insights into the immunopathogenesis of BVE. Collectively, the data indicate an imbalance characterised by robust activation of immune pathways in the presence of comparatively weak adaptive immune responses. Excessive innate immune activation may be associated with tissue injury and limited representation of effective immune control, in a setting where viral material remains detectable. In line with this, pathway analyses showed little overlap with transcriptional programs of lytic viral infections but strong similarity to signatures of non-lytic neurotropic viruses and autoimmune neuroinflammation. Notably, animal models have demonstrated that impaired or immature immune responses can prevent disease manifestation, highlighting the critical role of host immune regulation in BoDV-1 encephalitis. Together, these findings suggest that adaptive immune mechanisms may contribute more substantially to BoDV-1 disease pathogenesis rather than the response being exclusively innate and therefore warrant consideration in future therapeutic strategies.

## Supplementary Information

Supplementary Material 1.

## Data Availability

The datasets used and/or analysed during the current study are available from the corresponding author. The authors confirm that data supporting the findings of this study are also available in the Supplementary Material.

## Abbreviations

BoDV-1: Borna disease virus 1
BVE: Bornavirus encephalitis
C: Copies
FFPE: Formalin-fixed and paraffin-embedded
GSEA: Gene set enrichment analysis
HE: Hematoxylin and eosin
H-Score: Histopathological score
HSV-1: Herpes simplex virus 1
IHC: Immunohistochemistry
ISGs: Interferon-stimulated genes
KEGG: Kyoto encyclopedia of genes and genomes
MRI: Magnetic resonance imaging
NK: Natural killer
RT-qPCR: Quantitative reverse transcription polymerase chain reaction
RV: Rabies virus
