# Supplementary material for: Multimodal profiling of immune responses reveals innate-adaptive immune imbalance in human bornavirus encephalitis
Source: Acta Neuropathol Commun. 2026 May 14;14:109. doi: 10.1186/s40478-026-02319-6 (PMC13181928; doi:10.1186/s40478-026-02319-6)
Supplement: Supplementary file 1 — Supplementary Material 1. [file 40478_2026_2319_MOESM1_ESM.docx]

**Multimodal Profiling of Immune Responses Reveals Innate-Adaptive**

**Immune Imbalance in Human Bornavirus Encephalitis**

**Supplementary Material**

**Abbreviations**

BoDV-1= Borna disease virus 1; CT = cycle threshold; FFPE = formalin-fixed and paraffin-embedded; H-Score = histopathological Score; RT-qPCR = real-time quantitative polymerase chain reaction

**Material and Methods**

*Material*

Brain autopsy material from four individuals who died of Borna Disease Virus 1 (BoDV-1) encephalitis between 2022 and 2024 was included in this study. All cases comprised deceased female patients, with ages ranging from 39 to 71 years (median: 68.5 years). Cases 3 and 4 have been previously described [3, 10], whereas the other two cases have not been published yet.

Autopsy was performed after brain fixation in 4% buffered formalin. To analyse the viral distribution pattern in two planes, an alternative dissection protocol was developed for this study, in which one hemisphere was lamellated in sagittal sectioning (apart from Case 4) while the other hemisphere was subjected to coronal sectioning after transection of the hemispheres in the midline.

For all patients, complete cross-sections were embedded. For two cases (Case 1 and Case 2), a complete sagittal section of the right hemisphere and a complete coronary section of the left hemisphere was prepared. For Case 3, the right sagittal section was available; for Case 4 a coronary section through both hemispheres was conducted to assess the symmetry of viral distribution. The cross-sections were subdivided into approximately equally sized blocks and subsequently embedded into capsules, allowing later reconstruction of the original whole-brain slices. The tissue was again, fixed in 4% buffered formalin and embedded in paraffin resulting in formalin-fixed, paraffin-embedded (FFPE) material. In total, 157 FFPE blocks were included in the following analyses (median 37 blocks per case; range 31 to 52). Control tissue was obtained from a 64-year-old female individual who died of toxic cardiocirculatory failure in the setting of advanced metastatic breast carcinoma, combined with respiratory failure due to malignant pleural effusion. Two FFPE blocks, containing the left thalamus and frontal cortex, respectively, were selected as control regions for analysis.

Ethical approval for this study was granted by the ethics committee of the Ludwig-Maximilians-University ethics committee, responsible for Augsburg University Hospital (approval no. 23-0267).

*Immunohistochemistry*

Immunohistochemistry for BoDV-1, Iba-1, GFAP, CD3 and CD20 was performed using a fully automated staining system (Leica BOND RX, Leica Biosystems, Germany) on 2 µm thick slides that had been baked at 60°C for 60 minutes. Heat-induced epitope retrieval was performed for all antibodies in EDTA buffer at 100°C for 20 minutes. Thereafter, the tissue was incubated with the primary antibodies: Bo18 antibody against the nucleoprotein of BoDV-1 (monoclonal mouse antibody, dilution: 1:1000, as previously described [4, 5, 8, 11]); anti-Iba-1 (polyclonal, rabbit, dilution: 1:500, Fujifilm, Japan), anti-GFAP (monoclonal (SP78), rabbit, dilution: 1:500, Sigma-Aldrich, United States), anti-CD3 (monoclonal (2GV6), rabbit, ready-to-use; Roche Diagnostics, Switzerland) and anti-CD20 (monoclonal (L26), rabbit, ready-to-use; Roche Diagnostics, Switzerland). Antibody detection and counterstaining was performed through the BOND Polymer Refine Detection Kit (Leica Biosystems, Germany). For quality assurance, positive controls were included in each run.

*Digital Image Analysis*

All histological slides were digitised using the Pannoramic Scan II Digital Scanner (3DHISTECH, Hungary) equipped with the standard 20x/0.8 NA objective. Coronary and sagittal brain sections were subsequently digitally reconstructed in Power Point (Microsoft Corporation, United States) using screen shots of the whole slide images.

Quantitative image analysis was performed using the QuPath software [2]. For tissue sections stained with CD3, and CD20, positive cell detection algorithms were applied to quantify the number of immunoreactive cells within each tissue block (see Table 1). For Iba-1 and GFAP, the immunopositive area was measured and expressed as a percentage of the total tissue area. This approach was chosen because activation of astrocytes and microglia is accompanied by marked morphological transformations, including cellular hypertrophy and increased arborisation, which result in an expanded area of marker expression.

BoDV-1-positive cells were further analysed using the semi-quantitative histopathological (H)-Score approach implemented in QuPath. This score is calculated by multiplying the percentage of positive cells at each staining intensity (1 = weak, 2 = moderate, 3 = strong) by the corresponding intensity value, and summing the results to obtain a composite score per region [6, 9]. This method was specifically chosen for BoDV-1 because, unlike the other markers used primarily for cell type identification, the staining intensity of BoDV-1 provides additional information on the intracellular viral load, thereby allowing a more nuanced quantification of viral presence.

*QuPath-based parameters for positive cell detection*

| **QuPath-based Parameters** |
| --- |
| Nucleus Parameters:   - Background radius: 5.8 - Median filter radius: 3.4 - Sigma: 1.5 - Minimum area: 5 - Maximum area: 400 - Threshold: 0.07 - Maximum background intensity: 2   Cell Parameters:   - Cell expansion: 2.5   General Parameters:   - Smooth boundaries: enabled - Make measurements: enabled   Intensity Threshold Parameters (score based on Cell DAB optical density mean):   - 1+: 0.1536 - 2+: 0.2557 - 3+: 0.3471 |

***Supplementary Material Table 1*** *QuPath parameters for immunohistochemical positive cell detection.* *Finalised detection parameters used for QuPath-based positive cell detection in tissue blocks stained for BoDV-1, CD3, and CD20 are summarised in the table*

*BoDV-1 detection by Polymerase Chain Reaction*

BoDV-1 detection by real-time quantitative polymerase chain reaction (RT-qPCR) was conducted as reported previously [1]. In short, QiaAmp Viral RNA MiniKit (Qiagen, Germany) was used for RNA extraction from 2-3 10 μm thick sections of each FFPE block. Viral RNA amplification of the p24 (phosphoprotein) gene of BoDV-1 was quantified using cycle threshold (CT) values calculated from the second derivative of the amplification curve. BoDV-1 RNA copy numbers were determined by referencing CT values to a standard curve prepared from a synthetic positive control oligonucleotide, which was titrated to known concentrations and molarity. The limit of detection is 3.4 copies/reaction [1].

*RNA Extraction and Transcriptome-Based Analysis Using the Nanostring nCounter Technology*

Based on the distribution of viral load as measured by RT-qPCR, 12 regions/FFPE blocks per case were selected for transcriptome-based analysis of the neuroimmunological profile. RNA was isolated from 2-3 10 μm thick sections of each FFPE block using the Maxwell RSC RNA FFPE Kit (Promega Corporation, United States), following the manufacturer’s protocol. RNA concentration was measured using the Qubit RNA HS Assay Kit (Thermo Fisher Scientific, United States), and RNA integrity was assessed with the Agilent RNA 6000 Nano Kit on the Agilent 2100 Bioanalyzer (Agilent Technologies, United States). The RNA input concentration (5µl/50ng) was calculated for each sample and used for hybridisation in the NanoString nCounter gene expression assay, performed according to the manufacturer’s instructions Gene Expression CodeSet RNA Hybridisation Protocol as reported previously [7]. Gene expression was assessed using the NanoString Neuroinflammation Panel, which targets approximately 770 genes associated with neuroinflammatory processes, enabling comparison of immune profiles between brain regions with low, medium, and high viral loads.

*Statistical Analysis*

Transcriptome data generated using the NanoString nCounter platform were processed and analysed in Python (version 3.12.7, Python Software Foundation, United States), which was also employed for all statistical evaluations. Statistical significance was assessed at p < 0.05 and p <0.01. To investigate associations between variables, both Pearson’s product-moment correlation coefficient and Spearman’s rank correlation coefficient were calculated, thereby capturing both linear and monotonic relationships. For Case 4, the symmetry of viral load distribution was assessed by performing a paired t-test. For this purpose, quantitative viral load measurements were obtained from anatomically corresponding regions in the left and right hemispheric blocks and compared pairwise. Additionally, a Pearson correlation analysis was conducted to evaluate the strength and direction of the linear association between corresponding measurements from the left and right sides.

**Results**

*Regional Distribution and Quantitative Assessment of BoDV-1 Viral Loads*

|  |  | Case 1 | Case 2 | Case 3 | Case 4 |
| --- | --- | --- | --- | --- | --- |
| RNA (c/ng) | Mean | 94 998.83 | 9 343.39 | 462 711.72 | 170.37 |
|  | Median | 89 645.07 | 5 554.53 | 434 250.76 | 16.99 |
|  | Range | 13 379.58 - 226 269.20 | 733.95 - 44 465.29 | 130 041.15 - 1 024 811.20 | 1.56 - 1 411.11 |
| H-Score | Mean | 36.23 | 46.77 | 54.35 | 6.11 |
|  | Median | 35.22 | 43.48 | 51.06 | 3.12 |
|  | Range | 10.85 - 9.65 | 9.58 - 114.95 | 21.16 - 109.47 | 0.00 - 37.25 |

***Supplementary Material Table 2*** *Quantification of viral loads and H-Score values for individual cases, including corresponding mean values*

***
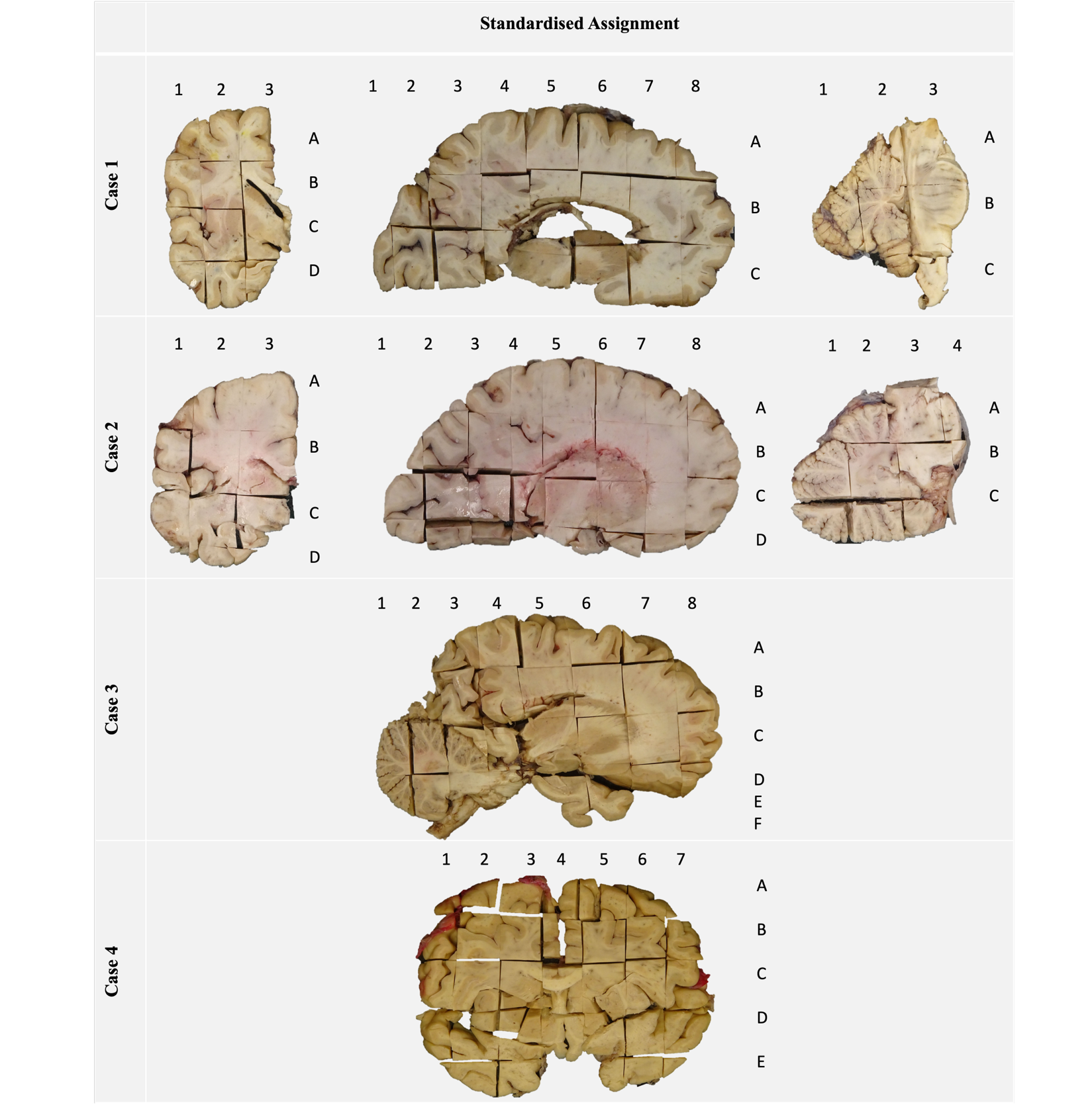
****General Standardised Anatomical Assignment of Tissue Blocks*

***Supplementary Material Figure 1*** *Standardised anatomical assignment of tissue blocks across four cases.* *For each case, coronal and/or sagittal brain sections are schematically depicted with consistent block labelling to document anatomical localisation. This representation allows reconstruction of sampling strategy and comparability across cases*

*Labelling, Viral Load and H-Score Values by Anatomical Block and Case*

| **Case 1** |  |  |
| --- | --- | --- |
| *Labelling*  A1 (coronary)  A2  A3  B1  B2  B3  C1  C2  C3  D1  D2  D3  A1 (sagittal)  A2  A3  A4  A5  A6  A7  A8  B1  B2  B3  B4  B5  B6  B7  B8  C1  C2  C3  C4  C5  C6  C7  C8  A1 (Cerebellum)  A2  A3  B1  B2  B3  C1  C2  C3 | *RNA (Copies per ng RNA)*  126 938.1  96 546.9  179 229.5  157 024.8  46 866.4  89 189.2  161 775.9  88 831.4  155 672.8  127 431.2  139 931.7  226 269.2  -  -  100 075.17  125 620.50  54 072.74  75 408.36  98 039.13  192 017.15  -  89 555.04  59 505.06  57 229.91  92 352.09  111 969.11  79 445.84  66 086.96  36 010.84  32 686.08  36 233.67  89 735.10  60 939.18  145 371.89  129 304.70  140 394.10  14 690.42  16 688.84  131 840.67  13 379.58  17 302.21  72 706.94  -  -  65 584.96 | *H-Score*  44.21  28.09  24.08  23.34  23.98  50.73  39.9  35.16  27.26  22.13  33.33  57.82  -  -  35.64  13.12  14.69  17.18  10.85  34.31  -  48.92  53.01  36.56  39.76  39.77  37.67  35.88  32.47  43.13  57.22  89.65  42.63  29.69  32.74  34.98  57.11  33.4  57.4  35.29  18.2  22.25  -  -  35.67 |
| **Case 2** |  |  |
| *Labelling*  A1 (coronary)  A2  A3  B1  B2  B3  C1  C2  C3  D1  D2  D3  A1 (sagittal)  A2  A3  A4  A5  A6  A7  A8  B1  B2  B3  B4  B5  B6  B7  B8  C1  C2  C3  C4  C5  C6  C7  C8  D1  D2  D3  D4  D5  D6  D7  D8  A1 (Cerebellum)  A2  A3  A4  B1  B2  B3  B4  C1  C2  C3  C4 | *RNA (Copies per ng RNA)*  12 227.07  8 265.58  20 000  9 186.09  6 699.03  26 637.68  19 295.39  10 227.27  26 608.70  15 054.64  35 555.56  44 465.29  -  9 184.99  3 195.70  8 018.87  5 518.21  7 110.44  5 910.36  17 596.90  6 300.11  1 374.07  1 439.53  2 363.95  1 631.88  4 364.66  2 929.64  7 932.10  2 211.81  1 396.04  2 068.38  4 347.83  3 060.98  2 632.08  6 287.88  4 523.81  3 336.81  3 583.33  4 833.84  5 371.05  8 868.50  10 041.27  16 904.76  -  -  4 828.66  3 904.76  20 648  733.95  2 019.23  3 300.97  42 332.42  2 067.94  1 867.28  5 590.85  - | *H-Score*  89.36  45.04  26.32  50.67  34.2  27.84  82.25  25.13  44.14  23.61  51.95  109.38  -  114.95  48.25  31.88  35.47  27.55  48.63  54.88  46.39  49.7  39.47  40.1  41.79  41.05  34.29  29.53  57.69  46.05  54.2  67.37  45.65  35.68  40.69  26.81  45.58  48.08  34  42.93  97.96  70.21  70.37  -  -  39.06  44.03  86.5  9.58  24.42  24.42  21.96  22.08  31.35  51.8  - |
| **Case 3** |  |  |
| *Labelling*  A1  A2  A3  A4  A5  A6  A7  A8  B1  B2  B3  B4  B5  B6  B7  B8  C1  C2  C3  C4  C5  C6  C7  C8  D1  D2  D3  D4  D5  D6  D7  D8  E3  F3 | *RNA (Copies per ng RNA)*  -  -  230 896.32  360 952.38  478 964.40  541 666.67  775 335.78  -  130 041.15  203 985.51  179 669.03  264 777.89  466 019.42  434 250.76  600 240.10  838 371.62  160 763.89  214 239.48  199 000  819 314.64  363 242.82  262 846.75  273 609.56  498 599.44  199 881.38  285 554.31  671 081.68  807 017.54  806 666.67  739 424.70  587 878.79  1 024 811.2  362 459.55  562 500 | *H-Score*  -  -  61.47  51.06  65.5  73.78  109.47  -  34.78  65.21  76.98  48.02  51.78  42.63  64.98  72.54  24.15  31.96  43.26  81.69  92.86  37.36  41.17  30.95  27.58  21.16  64.55  71.29  29.69  66.52  47.53  91.49  29.29  34.3 |
| **Case 4** |  |  |
| *Labelling*  A1  A2  A3  A4  A5  A6  A7  B1  B2  B3  B4  B5  B6  B7  C1  C2  C3  C4  C5  C6  C7  D1  D2  D3  D4  D5  D6  D7  E1  E2  E3  E5  E6  E7 | *RNA (Copies per ng RNA)*  7.73  1.56  7.61  8.42  10.59  10.76  15.14  11.02  7.29  16.99  13.89  10.02  6.66  18.08  5.68  46.93  84.6  68.51  326.62  14.44  13.82  18.35  513.3  582.8  243.97  730.158  270.22  13.04  20  33.14  1 038.6  1 411.1  40.98  NEG | *H-Score*  1  4.36  4.6  2.01  4.59  1.58  0.49  0.86  2.34  3.04  9.87  9.39  8.73  3.20  0.16  2.81  13.77  2.14  37.25  3.26  0  1.88  13.87  17.75  13.81  18.11  5.03  0.78  0.81  2.23  8.37  8.47  0.57  0.55 |

***Supplementary Material Table 3*** *Viral load quantification and immunohistochemical scoring across four cases.* *For each case, FFPE tissue blocks are listed by anatomical labelling. RNA-based viral load measurements (copies per ng RNA) and corresponding semi-quantitative immunohistochemical H-Scores are provided for each block*

*
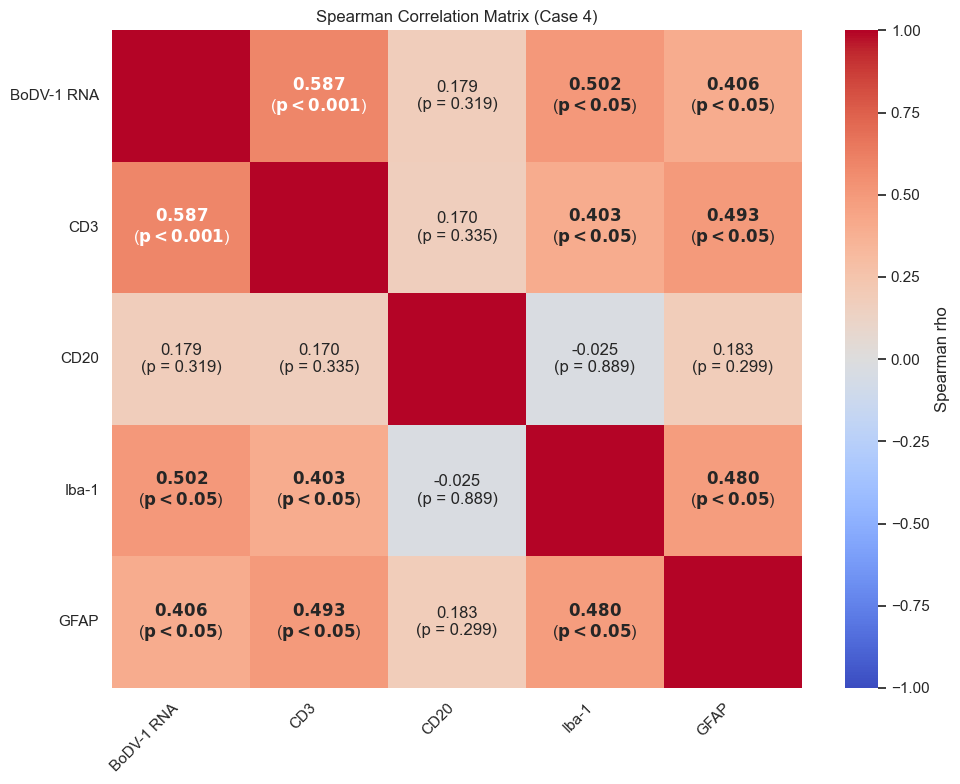
Correlation Analysis Identifies Coordinated T Cell, Microglial, and Astrocytic Responses to BoDV-1 RNA Levels*

***Supplementary Material Figure 2*** *Spearman correlation analysis among viral load (BoDV-1 RNA) and inflammatory markers CD3, CD20, Iba-1 and GFAP (Case 4). Significant values are marked in bold*

*Immunohistochemical findings in BoDV-1 encephalitis in Case 4*

*****Supplementary Material Figure 3*** *Immunohistochemical characteristics of BoDV-1 encephalitis in Case 4.* ***(A.)*** *BoDV-1 staining demonstrating viral presence within the affected tissue. Scale bar 50μm.* ***(B.)*** *CD3 illustrating T cell infiltration with perivascular accentuation as well as diffuse distribution into the parenchyma. Scale bar 100μm.* ***(C.)*** *CD20 marking B cells, which are predominantly localised in perivascular regions without significant parenchymal infiltration. Scale bar 50 μm,* ***(D.)*** *GFAP demonstrating marked astroglial activation (atrogliosis). Scale bar 50 μm.* ***(E.)*** *Iba-1 highlighting activated microglia with formation of microglial nodules. Scale bar 100μm*

*******Transcriptomic Immune Cell Mapping Suggests Viral Load-dependent Modulation of Immune and Glial Populations*

 ***Supplementary Material Figure 4*** *Cellular landscape across BoDV-1 viral load groups. Distribution of immune cell populations* ***(A.)*** *across 50 brain tissue samples, stratified by BoDV-1 load (low, medium, high) and controls. Cell-type composition was inferred from transcriptomic data to assess viral load-dependent neuroinflammatory changes. Subtype distributions of T cells* ***(B.)****, astrocytes* ***(C.)****, and microglia* ***(D.)*** *are also shown*

***Supplementary Material Figure 5*** *Cellular landscape across BoDV-1 viral load groups for Case 3. Distribution of immune cell populations* ***(A.)****, stratified by BoDV-1 load (low, medium, high) and controls. Subtype distributions of T cells* ***(B.)****, astrocytes* ***(C.)****, and microglia* ***(D.)*** *are also shown*

*Correlation analysis between immunohistochemical quantification and nCounter gene expression data for different cellular markers*

***
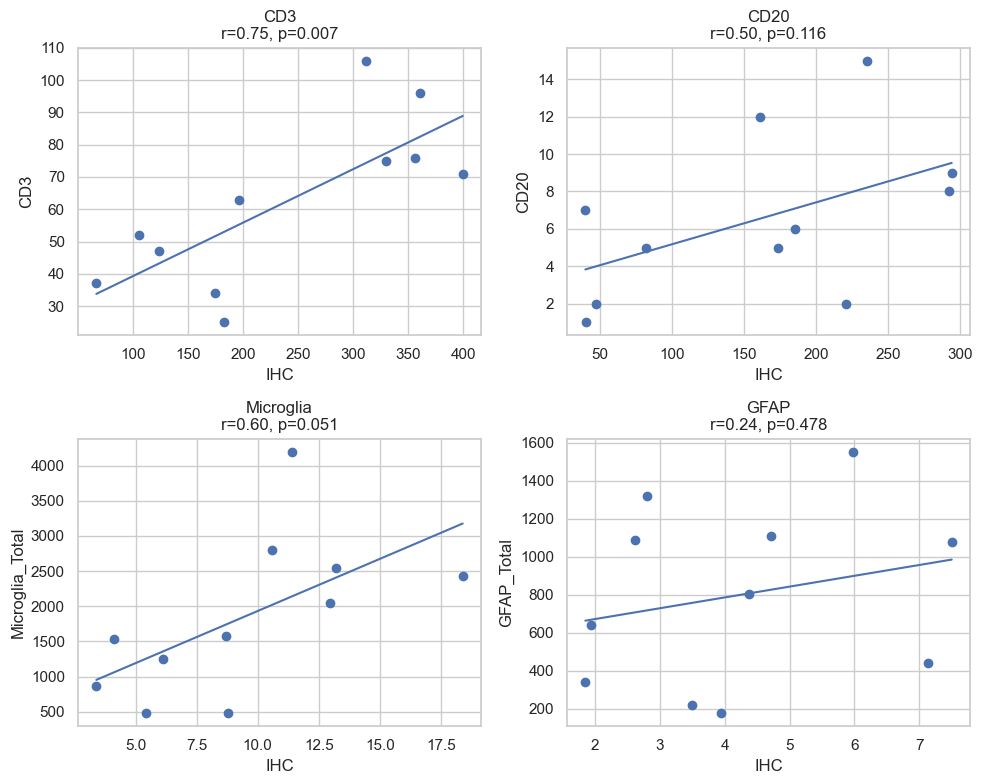
Supplementary Material Figure 6*** *Correlation of immunohistochemical quantification with nCounter gene expression data across different cellular markers. A strong and significant positive correlation was observed for CD3. Microglia-associated markers showed a moderate correlation. In contrast, CD20 and GFAP did not demonstrate statistically significant correlations. Each dot represents an individual sample from Case 4, with linear regression lines shown for visualisation*

*Pathway and Pseudotime Analysis Reveal Load-dependent Innate Activation and Limited Adaptive Immune Engagement*

******

***Supplementary Material Figure 7*** *Pathway-specific immune activation across BoDV-1 viral load groups. Bar plot showing total gene expression counts for selected immune-related pathways in samples stratified by BoDV-1 viral load groups across all cases* ***(A.)*** *and for Case 3* ***(B.)***

*Gene Expression Analysis Shows Threshold-dependent Activation of Innate and Inflammatory Pathways*

***Supplementary Material Figure 8*** *Differential gene expression in BoDV-1-infected brain regions across viral load stages and in comparison, to non-infected control tissue in Case 3.* *Volcano plots pairwise comparisons between brain regions with differing BoDV-1 viral loads and control tissue in Case 3. Genes significantly upregulated (red) or downregulated (blue) are highlighted according to significance thresholds (p < 0.05 and p < 0.01)*

**References**

1 Allartz P, Hotop SK, Muntau B, Schlaphof A, Thomé-Bolduan C, Gabriel M, Petersen N, Lintzel M, Behrens C, Eggert Pet al (2024) Detection of bornavirus-reactive antibodies and BoDV-1 RNA only in encephalitis patients from virus endemic areas: a comparative serological and molecular sensitivity, specificity, predictive value, and disease duration correlation study. Infection 52: 59-71 Doi 10.1007/s15010-023-02048-1

2 Bankhead P, Loughrey MB, Fernández JA, Dombrowski Y, McArt DG, Dunne PD, McQuaid S, Gray RT, Murray LJ, Coleman HGet al (2017) QuPath: Open source software for digital pathology image analysis. Scientific Reports 7: 16878 Doi 10.1038/s41598-017-17204-5

3 Bayas A, Menacher M, Lapa C, Tappe D, Maurer C, Liesche-Starnecker F, Schneider H, Naumann M (2024) 18fluorodeoxyglucose PET/CT as possible early diagnostic tool preceding MRI changes in Borna disease virus 1 encephalitis. Lancet (London, England) 403: 665–666 Doi <https://doi.org/10.1016/S0140-6736(24)00049-7>

4 Haas B BH, Rott R. (1986) Purification and properties of an intranuclear virus-specific antigen from tissue infected with Borna disease virus. J Gen Virol: 235-241

5 Herden C, Schluesener HJ, Richt JA (2005) Expression of allograft inflammatory factor-1 and haeme oxygenase-1 in brains of rats infected with the neurotropic Borna disease virus. Neuropathol Appl Neurobiol 31: 512-521 Doi 10.1111/j.1365-2990.2005.00668.x

6 Kraus JA, Dabbs DJ, Beriwal S, Bhargava R (2012) Semi-quantitative immunohistochemical assay versus oncotype DX(®) qRT-PCR assay for estrogen and progesterone receptors: an independent quality assurance study. Mod Pathol 25: 869-876 Doi 10.1038/modpathol.2011.219

7 Kulkarni MM (2011) Digital multiplexed gene expression analysis using the NanoString nCounter system. Curr Protoc Mol Biol Chapter 25: Unit25B.10 Doi 10.1002/0471142727.mb25b10s94

8 Liesche F, Ruf V, Zoubaa S, Kaletka G, Rosati M, Rubbenstroth D, Herden C, Goehring L, Wunderlich S, Wachter MFet al (2019) The neuropathology of fatal encephalomyelitis in human Borna virus infection. Acta neuropathologica 138: 653–665 Doi <https://doi.org/10.1007/s00401-019-02047-3>

9 Meyerholz DK, Beck AP (2018) Principles and approaches for reproducible scoring of tissue stains in research. Lab Invest 98: 844-855 Doi 10.1038/s41374-018-0057-0

10 Vollmuth Y, Jungbäck N, Mögele T, Schmidt-Graf F, Wunderlich S, Schimmel M, Rothe C, Stark L, Schlegel J, Rieder Get al (2024) Comparative Study of Virus and Lymphocyte Distribution with clinical Data suggests early high dose Immunosuppression as potential Key Factor for the Therapy of Patients with BoDV-1 Infection. Emerging microbes & infections: Doi <https://doi.org/10.1080/22221751.2024.2350168>

11 Werner-Keiss N, Garten W, Richt JA, Porombka D, Algermissen D, Herzog S, Baumgärtner W, Herden C (2008) Restricted expression of Borna disease virus glycoprotein in brains of experimentally infected Lewis rats. Neuropathol Appl Neurobiol 34: 590-602 Doi 10.1111/j.1365-2990.2008.00940.x
